# A Multiscale Denoising Framework Using Detection Theory with Application to Images from CMOS/CCD Sensors

**DOI:** 10.3390/s19010206

**Published:** 2019-01-08

**Authors:** Khuram Naveed, Shoaib Ehsan, Klaus D. McDonald-Maier, Naveed ur Rehman

**Affiliations:** 1 Department of Electrical and Computer Engineering, COMSATS University, Park Road, Islamabad 45550, Pakistan; naveed.rehman@comsats.edu.pk; 2 School of Computer Science and Electronic Engineering, University of Essex, Colchester CO4 3SQ, UK; sehsan@essex.ac.uk (S.E.); kdm@essex.ac.uk (K.D.M.-M.)

**Keywords:** multiscale, Gaussian and Poisson denoising, CMOS/CCD image sensors, detection theory, binary hypothesis testing, variance stability transformation (VST)

## Abstract

Output from imaging sensors based on CMOS and CCD devices is prone to noise due to inherent electronic fluctuations and low photon count. The resulting noise in the acquired image could be effectively modelled as signal-dependent Poisson noise or as a mixture of Poisson and Gaussian noise. To that end, we propose a generalized framework based on detection theory and hypothesis testing coupled with the variance stability transformation (VST) for Poisson or Poisson–Gaussian denoising. VST transforms signal-dependent Poisson noise to a signal independent Gaussian noise with stable variance. Subsequently, multiscale transforms are employed on the noisy image to segregate signal and noise into separate coefficients. That facilitates the application of local binary hypothesis testing on multiple scales using empirical distribution function (EDF) for the purpose of detection and removal of noise. We demonstrate the effectiveness of the proposed framework with different multiscale transforms and on a wide variety of input datasets.

## 1. Introduction

Digital images acquired using complementary metal oxide semiconductors (CMOS) or charged coupled devices (CCD) image sensors are subject to noise from two notable sources, i.e., electronic instruments and the photo-sensing devices [1,2]. This noise is typically modelled using a mixture of Poisson and Gaussian distributions, namely, Poisson–Gaussian distribution [3,4]. In cases where the Poisson component of the noise is dominant, the Gaussian component is ignored and noise is modelled using the Poisson distribution [5,6].

For the purpose of denoising, estimation of noise parameters of the signal-dependent noise from CMOS/CCD sensors is a problem of interest [7,8]. A mixed Poisson–Gaussian distribution was used to model the practical sensor noise which was subsequently used for denoising [9]. In [10], Poisson statistics in combination with maximum likelihood estimation are used to restore images from optic acquisition systems. A Bayesian framework is developed for denoising and deconvolution of Poisson–Gaussian noise [11]. In addition, a post processing technique for Poisson denoising using best linear prediction on the local image patches is introduced in [12].

Similar to the Stein’s unbiased risk estimator (SURE) which is an estimate of the mean squared error (MSE) for Gaussian noise [13], a Poisson unbiased risk estimator (PURE) is estimated and used with the linear expansion techniques (LET) to formulate a state-of-the-art Poisson denoising method known as *PureLet* [14]. This technique was also extended for Poisson–Gaussian denoising, whereby Poisson–Gaussian risk estimate (PGURE) was minimized [15]. A Purelet deconvolution technique has also emerged for restoring images in the presence Poisson–Gaussian noise [16]. Furthermore, *PURE* estimate has also been employed for extension of the non-local mean (NLM) filtering to Poisson denoising, which we call *Poiss-NLM* [17]. A non-local application of principle component analysis (NL-PCA) for Poisson denoising has been achieved by patch-ization of the noisy image [18].

An alternate approach to the problem involves ‘Gaussianization’ of Poisson noise through variance stability transformation (VST), followed by traditional noise filtering methods [19]. In this class of methods, first, VST is applied on a low (photon) count image, resulting in transformation of Poisson noise to approximately Gaussian noise which is not only signal independent but also has constant variance. In this way, a Poisson denoising problem can be modeled as a Gaussian denoising problem post VST. As a result, Gaussian denoising methods can be used for suppressing noise, followed by the inverse VST (IVST) to obtain the Poisson denoised image.

Poisson denoising using Anscombe variance stability transformation (AT) [20] has been studied in [21,22,23], which involves state-of-the-art Gaussian denoising methods. However, inverse Anscombe transformation does not perform the exact reconstruction due to a bias at low photon count, which has been empirically improved in [22]. Similarly, VST has also been used for removing/reducing Poisson–Gaussian noise in [24] where generalized Anscombe transformation (GAT) [25] is used for approximate Gaussian transformation of Poisson–Gaussian noise. This is followed by the use of Gaussian denoising methods such as BLSGSM (introduced in [26]). Finally, inverse GAT transformation is performed to yield the denoised image.

A multiscale extension of VST (MS-VST) is employed for removing mixture of the Poisson–Gaussian noise from medical images in [27]. A similar approach using MS-VST is also devised for Poisson denoising in [28]. Other multiscale approaches for Poisson denoising employ hypothesis testing using multiscale Haar transform [29,30].

In this work, we propose a framework for denoising data corrupted with Poisson or mixed Poisson–Gaussian noise, which is found in images obtained from CMOS or CCD image sensors. The proposed framework uses hypothesis testing framework, typically used in signal detection theory, at multiple data scales and employs test statistics based on empirical distribution function (EDF), e.g., AD and CVM statistics. While our previous work used goodness-of-fit (GoF) test based hypothesis testing for Gaussian denoising of 1D (time series) [31,32,33] and 2D data (images) [34,35], the novel contributions in this work are as follows: (i) the proposed framework caters for both Poisson and mixed Poisson–Gaussian noise from CMOS/CCD sensors; (ii) the proposed framework is generalized in a sense that it has been implemented by using linear and non-linear multiscale transform domain methods; (iii) complete theoretical and mathematical framework of the proposed methodology is presented in the context of detection theory; (iv) extensive results on images corrupted with non-Gaussian noise have been included with special emphasis on images obtained from CMOS/CCD sensors.

The paper is arranged as follows: Section 2 gives an insight into CMOS imaging and noise models while statement of the problem is given in Section 3. A review of the signal detection theory is presented in Section 4. Section 5 presents the proposed non-Gaussian denoising framework while the discussion on experimental results is presented in Section 6. Section 7 concludes the paper with a discussion on avenues for future work.

## 2. CMOS Imaging and Sources of Noise

**Figure 1:**
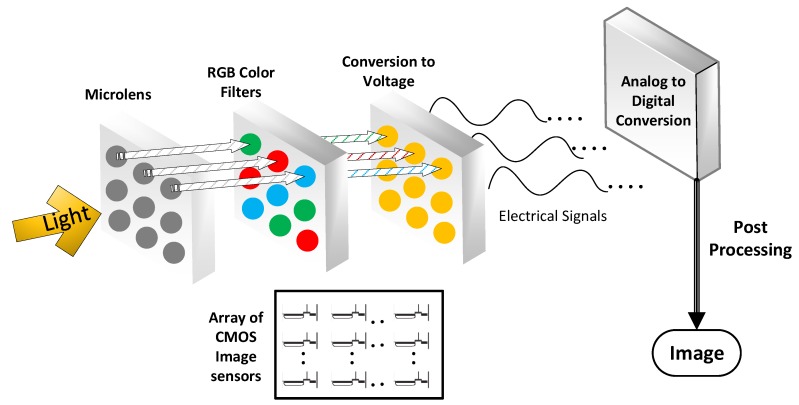
Pipeline of CMOS imaging acquisition system (adapted from [38]).

Recent progress in the design of CMOS imaging technology has led to extensive use of image acquisition systems in real world applications, i.e., installation of cameras in mobile phones, tablets, personal computers etc. A CMOS imaging system is essentially composed of two dimensional array of CMOS sensors each of which produces a pixel value [36,37]. The architecture of the CMOS image acquisition system is given in Figure 1 whereby a pipeline of modules required to capture an image is shown. To begin with, a micro-lens array is installed to increase the concentration of light incident on the photo-detector. This is followed by a color filter array which generates only one of the red, green or blue signal at each pixel. Next, an array CMOS imaging sensors capture the analog signals and converts them into electrical signals which are subsequently digitized using the analog to digital converters to generate pixel values. Finally, post processing operation is performed to adjust white balancing and perform color correction [38].

**Figure 2:**
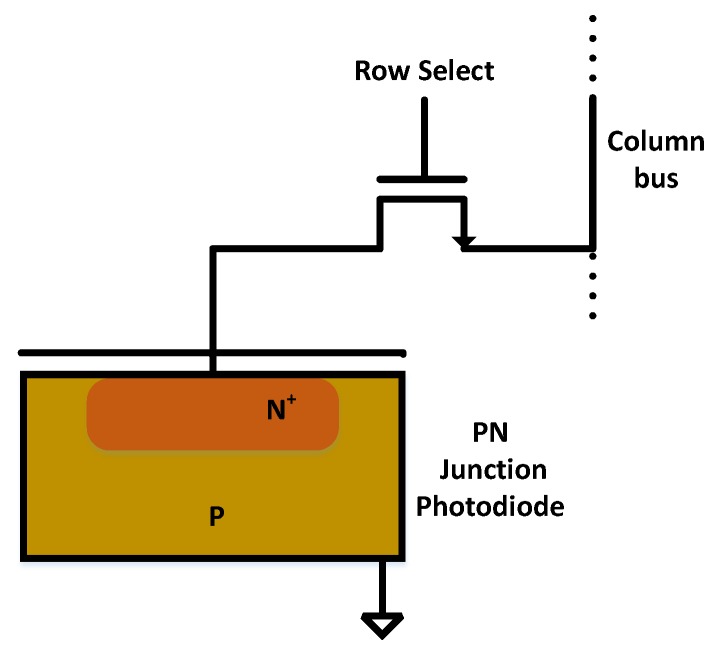
CMOS photo-sensing pixel architecture (adapted from [41]).

**Figure 3:**
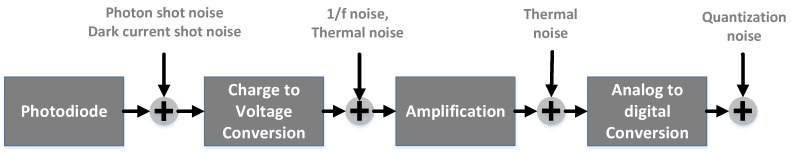
Noise model of CMOS image sensor (adapted from [42]).

While CMOS image sensing technology is revolutionizing the digital imaging by shrinking the pixel pitch [39], one of the major challenges includes reducing image noise at the time of acquisition [40]. In the case of CMOS imaging sensors, main sources of noise include sensor electronics and photon starvation. For precise modeling of noise in the CMOS sensors, it is important to look into the pixel sensing architecture shown in Figure 2. The pixel circuit is composed of a photodiode and a switching transistor. The imaging principle works as follows: During exposure, photons fall on the reverse biased photodiode leading to the decrease of the reverse voltage across the diode. Subsequently, voltage across photodiode is measured or read at the end of the exposure and photodiode is reset for another exposure [41]. This is known as passive pixel model while the active CMOS pixel model includes the amplification of the read signal which not only increases the sensitivity of the CMOS sensor but also helps reduce the noise [41]. Figure 3 gives an account of various types of noise corrupting the CMOS sensed image.

The quantum nature of light dictates that the amount of photons incident on the photodiode is never a certainty. This fluctuation in photon count results in shot noise in the acquired image [41]. Similarly, dark current non-uniformity is an exposure dependent fixed pattern noise which also has a temporal noise associated with itself, known as dark current shot noise. Noise due to photon fluctuations are signal-dependent and are modelled using Poisson distribution [42], where the mean and the variance of the Poisson process equals the signal strength.

Moreover, charge to voltage conversion and subsequent amplification of the electrical signal are also noisy owing to electronic fluctuations. These processes notably introduce flicker or 1/f noise and thermal noise, which are modelled using the additive white Gaussian noise (AWGN) [42]. In addition, residual error due to quantization is also modelled using the independent additive white Gaussian noise [15].

Based on the above discussion, noise due to CMOS image sensors is typically modelled using the mixture of Poisson and Gaussian distributions also termed as Poisson–Gaussian distribution [15,16,42]. However, in situations of poor illumination or low light conditions noise may be dominantly Poisson distributed [14,20] since the effect of AWGN can be neglected due to low photon count in such cases.

## 3. Statement of Problem

Let $z_{i}$ denotes the pixels of the acquired noisy image Z using a CMOS sensor, which may be mathematically modelled as

(1) $$z_{i}=s_{i}+\eta_{i}^{p}\left(s_{i}\right)+\eta_{i}^{g},$$

where $s_{i}$ denotes expected pixels of true image S, $\eta_{i}^{g}∼N\left(0,\sigma^{2}\right)$ denote the additive white Gaussian noise (AWGN) with zero mean and arbitrary variance $\sigma^{2}$ and $\eta_{i}^{p}\left(s_{i}\right)∼P\left(z_{i}|s_{i}\right)$ denotes the signal-dependent Poisson noise. Here, Poisson distribution $P(z_{i}|s_{i})$ is given as follows

(2) $$P\left(z_{i}|s_{i}\right)=\frac{s_{i}^{z_{i}}e^{-s_{i}}}{z_{i}!},$$

where ! denotes the factorial operation. Note that the vector i denotes pixel location, i.e., row and column indexes.

Under various physical limitations, i.e., low light or short exposure time, the effect of Gaussian noise may be neglected due to relative strength of the signal-dependent noise. In this case, the acquired pixel model (1) reduces to

(3) $$z_{i}=s_{i}+\eta_{i}^{p}\left(s_{i}\right).$$

In this case, $z_{i}$ will also be distributed by $P(z_{i}|s_{i})$ with a non zero mean $E\left[z_{i}|s_{i}\right]=s_{i}$. Hence, it can be concluded that the mean and variance of $\eta_{i}^{p}\left(s_{i}\right)$ are given as follows

(4) $$\begin{array}{c} E[\eta_{i}^{p}\left(s_{i}\right)]=0,\\ var\left[\eta_{i}^{p}\left(s_{i}\right)\right]=s_{i}. \end{array}$$

This means that the variance of each noise coefficient $\eta_{i}^{p}\left(s_{i}\right)$ is dependent on the corresponding true signal value $s_{i}$ and is parametrized by the peak value of the signal.

## 4. Preliminaries: Introduction to Hypothesis Testing in Detection Theory

**Figure 4:**
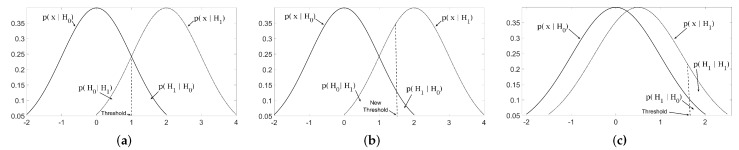
Depiction of a simple detection problem where the probability of null hypothesis $p\left(x|H_{0}\right)=N\left(0,\sigma^{2}\right)$ and probability of alternate hypothesis $p\left(x|H_{1}\right)=N\left(\mu ,\sigma^{2}\right)$ are plotted together. Here, (**a**) show the relationship between error probability regions $p(H_{0}|H_{1})$, $p(H_{1}|H_{0})$ and the detection probability region $p(H_{1}|H_{1})$; (**b**,**c**) highlight the trade off between $P_{fa}$ and $P_{d}$ with an increase in threshold value λ.

Classical detection theory [43] based on hypothesis testing assumes prior distribution models for signal and noise where the aim is to detect signal (with or without an additive noise part) while avoiding noise, e.g., communication channel, radar signal processing etc. A detection problem using hypothesis testing comprises of (i) null hypothesis $H_{0}$ of *noise only* case and (ii) alternate hypothesis $H_{1}$ concerning with *signal plus noise* detection. A classical example may be the detection of signal in a transmission medium where the noise is assumed to be distributed by zero mean Gaussian distribution $N(0,\sigma^{2})$ and *signal plus noise* is modeled by a non-zero mean Gaussian distribution $N(\mu ,\sigma^{2})$ as signal values when added to zero mean noise, contribute a mean μ to the distribution. Mathematically, this detection problem may be modeled as

(5) $$\begin{array}{c} H_{0}:x\in N\left(0,\sigma \right),\\ H_{1}:x\in N\left(\mu ,\sigma \right), \end{array}$$

where $H_{0}$ and $H_{1}$ denote the null and the alternate hypothesis, respectively, while *x* denotes an arbitrary value from the noisy signal x. Figure 4a plots probability distribution functions of each hypothesis, i.e., $p\left(x|H_{0}\right)=N\left(0,\sigma^{2}\right)$ and $p\left(x|H_{1}\right)=N\left(\mu ,\sigma^{2}\right)$. The problem here is to differentiate between the following hypothesis

(6) $$\begin{array}{c} H_{0}:x=\eta ,\\ H_{1}:x=s+\eta , \end{array}$$

where *s* denotes an arbitrary value from the true signal s and η denotes an arbitrary value from η distributed by $N(0,\sigma^{2})$.

Here, the decision in Equation (6) can be taken by comparing the observations *x* against a threshold λ. This is elaborated graphically in Figure 4a, where the threshold is plotted as a dotted line. Note that the values greater than the threshold $\lambda_{k}$ are more likely to be distributed according to $p(x|H_{1})$ while the values less then the threshold $\lambda_{k}$ are more likely to be distributed in accordance with $p(x|H_{0})$. Therefore, the above hypothesis testing problem can be given as

(7) $$\begin{array}{c} H_{0}:x\leq \lambda ,\\ H_{1}:x>\lambda . \end{array}$$

A trivial choice of threshold may be the point of intersection of $p(x|H_{0})$ and $p(x|H_{1})$ as shown in Figure 4a while a different choice of threshold is depicted in Figure 4b.

However, this kind of detector makes two types of errors: (type I) detecting $H_{0}$ when $H_{1}$ is given; and (type II) detecting $H_{1}$ when $H_{0}$ is given $p(H_{1}|H_{0})$. Figure 4 shows probability regions $p(H_{0}|H_{1})$ and $p(H_{1}|H_{0})$ corresponding to type I and II errors respectively, in the context of a simple detection problem. The type II error is also known as *false alarm* and the probability $p(H_{1}|H_{0})$ is termed as the *probability of false alarm* $\left(P_{fa}\right)$. Minimizing both errors simultaneously is not possible as decreasing one increases the other, however, these errors can be traded off for each other by adjusting the value of threshold λ, (as depicted by different choices of threshold in Figure 4). Typically, it is required to keep $P_{fa}$ very low in order to avoid the severe consequences of noise detected as signal. Hence, $P_{fa}$ is fixed to a very small value α to estimate a suitable threshold using the following relation

(8) $$P_{fa}=p\left(H_{1}|H_{0}\right)=Prob\left(x>\lambda \;|H_{0}\right)=\int_{\left\{x\;|\;x>\lambda \right\}}p\left(x|H_{0}\right)dx=\alpha ,$$

where the range {x|x>λ} denote the values *x* detected as signal (i.e., $H_{1}$) and Prob(·) denotes the probability of the given event and α is of the order of $10^{-3}$–$10^{-6}$.

On the other hand, the probability of the first type of error $p(H_{0}|H_{1})$ is minimized which in turn maximizes the probability of signal detection when signal is present, i.e., $p\left(H_{1}|H_{1}\right)=1-p\left(H_{0}|H_{1}\right)$, see Figure 4c. The probability of true signal detections $p(H_{1}|H_{1})$, also termed as probability of detection ($P_{d}$), is mathematically given as

(9) $$P_{d}=p\left(H_{1}|\;H_{1}\right)=Prob\left(x>\lambda \;|\;H_{1}\right)=\int_{\left\{x\;|\;x>\lambda \right\}}p\left(x|\;H_{1}\right)dx.$$

In detection theory, $P_{d}$ is required to be maximized with the minimization of $P_{fa}$.

The binary hypothesis defined in Equation (7) directly compares the data *x* against a threshold. More generally, some metric S(x) on *x* is used for this purpose, e.g., Neyman Pearson optimal detector [44] uses a statistic $S\left(x\right)=\frac{p(x|H_{1})}{p(x|H_{0})}$ to compare against the threshold λ for hypothesis testing as follows

(10) $$\begin{array}{c} H_{0}:S\left(x\right)\leq \lambda ,\;\;\;\;i.e.,\;\;\;\;x\in \eta ,\\ H_{1}:S\left(x\right)>\lambda ,\;\;\;\;i.e.,\;\;\;\;x\in s+\eta , \end{array}$$

where the distributions of null hypothesis $p(x|H_{0})$ and alternate hypothesis $p(x|H_{1})$ must be known a priori. Consequently, definition of $P_{fa}$ in the presence of test statistic S(x) changes to the following

(11) $$P_{fa}=Prob\left(S\left(x\right)>\lambda \;|H_{0}\right)=\int_{\left\{x\;|\;S(x)>\lambda \right\}}p\left(x|H_{0}\right)dx,$$

where {x|S(x)>λ} is composed of the values *x* for which S(x)>λ and as a consequence, threshold λ may be estimated by fixing the $P_{fa}=\alpha$. Similarly, $P_{d}$ changes to the following

(12) $$P_{d}=Prob\left(S\left(x\right)>\lambda \;|H_{1}\right)=\int_{\left\{x\;|\;S(x)>\lambda \right\}}p\left(x|H_{1}\right)dx.$$

A popular approach in detection theory is based on goodness-of-fit (GoF) test in which the test statistic S(x) is based on the information regarding the empirical distribution model of the data at hand. This approach also avoids the need to assume a prior distribution model for alternate hypothesis, since prior knowledge of the null distribution is adequate for binary hypothesis testing in Equation (7). Under such conditions, the test statistic S(x) estimates the distance between the empirical distribution function (EDF) $F\left(t\right)=\sum_{t}1.\left(x>t\right)$ of noisy observations x, and the null cumulative distribution function (CDF) $F_{0}\left(t\right)=\int_{t}p\left(x|\;H_{0}\right)dx$, where *t* is the support vector. There are a number of test statistics/measures used as detectors within the framework of the GoF test, but Anderson Darling (AD) statistic [45], Cramer Von Mises (CVM) statistic [46] are frequently used in detection problems [47,48], and are given, respectively, as follows

(13) $$S_{AD}\left(x\right)=\int_{-\infty }^{\infty }{\left(F_{0}\left(t\right)-F\left(t\right)\right)}^{2}\psi \left(F_{0}\left(t\right)\right)d\left(F_{0}\left(t\right)\right).$$

(14) $$S_{CVM}\left(x\right)=\int_{-\infty }^{\infty }{\left(F_{0}\left(t\right)-F\left(t\right)\right)}^{2}d\left(F_{0}\left(t\right)\right).$$

## 5. Proposed Denoising Framework Using Detection Theory

In this section, we propose a denoising framework to remove Poisson and Poisson–Gaussian distributed arising due to the CMOS/CCD image sensors. For this purpose, we first employ variance stability transformation (VST) to ‘Gaussianize’ the noise present in the CMOS/CCD images. Following that, image denoising is formulated as a detection problem whereby local hypothesis testing based on empirical distribution function (EDF) is employed.

Since, detection theory is employed on time series data [47,48], formulation of detection problem based on local EDF statistics for spatio-temporal data (images) in our case requires following notable adjustments.

1. To ensure the preservation of spatio-temporal characteristics of multiscale coefficients of noisy image, two dimensional (2D) windows of size l×l are considered around the coefficient for local hypothesis testing.
2. Two dimensional EDFs are not unique and are computationally expensive [49], therefore, their use for GoF testing on 2D data is not suitable. Consequently, in our work, we list the coefficients in the windows as 1D vectors followed by the computation of their unique (1D) EDF. Note that listing of 2D segments as 1D vectors is a common practice in image denoising methods whereby multivariate statistical distributions are used to model multiscale dependencies [50].

**Figure 5:**
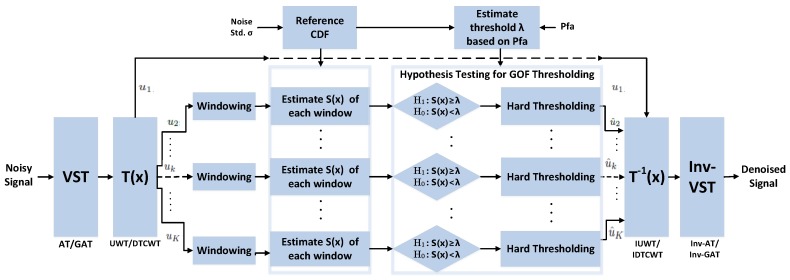
Block diagram of the proposed framework for Poisson and Poisson–Gaussian denoising using detection theory.

The block diagram of the proposed method is shown in Figure 5. The method involves VS transformation followed by multiscale hypothesis testing of data at local level using EDF statistics. The following subsections illustrate the main steps of the proposed framework.

### 5.1. Variance Stability Transform (VST)

We propose to use VST as a preprocessing step in the case of denoising images corrupted by Poisson and Poisson–Gaussian noise. Following the preprocessing step, the noise is effectively transformed into an independent Gaussian noise with constant variance which can be handled through a Gaussian denoising framework. For Poisson image pixels $z_{i}\in Z$, the AT [20] could be used for variance stabilization of signal-dependent noise as follows,

(15) $$x_{i}=2\sqrt{z_{i}+3/8}.$$

For variance stabilization of mixed Poisson–Gaussian noisy image Z, generalized Anscombe transformation (GAT) [25] is used

(16) $$x_{i}=\left\{\begin{array}{cc} \frac{2}{\alpha }\sqrt{\alpha z_{i}+\frac{3}{8}\alpha^{2}+\sigma^{2},} & \;z_{i}>-\frac{3}{8}\alpha -\frac{\sigma^{2}}{\alpha },\\ 0 & \;z_{i}\leq -\frac{3}{8}\alpha -\frac{\sigma^{2}}{\alpha }, \end{array}\right.$$

where the problem (1) is now transformed to Gaussian denoising problem since $x_{i}\in X$ in Equations (15) and (16) are pixels of the variance stabilized image corrupted with approximate Gaussian noise. Note that, GAT is a generalization of AT, as for scaling factor α=1 and σ=0 (i.e., absence of Gaussian noise), Equation (16) reduces to Equation (15).

### 5.2. Multiscale Local Hypothesis Testing Based on EDF

Typically, in detection problems, we are mainly interested in the detection of signal at a particular time (with or without additive noise part). In signal denoising, on the other hand, we are interested in separating signal from noise so that the effect of noise could be cancelled from the output data. That requires a modification in the classical binary hypothesis testing framework to be applied for denoising applications. In the denoising problem, the alternate hypothesis must correspond to the *signal only* case whereas the null hypothesis corresponds to the *noise only* case as before. To achieve that, we propose to apply the modified hypothesis testing within the framework of goodness-of-fit (GoF) test at multiple scales obtained via a multiscale transform.

Let T(·) denote a multiscale transform which decomposes a noisy signal x into multiscale coefficients $u_{k}^{\left(i\right)}$ at scale *k* and location *i*, as given below

(17) $$u_{k}^{\left(i\right)}=T\left(x\right).$$

For multiscale coefficients $u_{k}^{\left(i\right)}$ to either correspond to the true signal (only) or the noise (only), the T(·) must fulfill the following conditions:

1. T(·) must decompose a signal across multiple scales.
2. Across each scale, signal and noise must be distributed among separate coefficients/values.

Set of transform domain methods fulfilling the above conditions may include DWT and its redundant variants like DDDWT [51], DT-CWT [52] and UWT [53] etc.

Given that T(·) fulfills aforementioned conditions, we propose to formulate the *denoising* problem as a transformed hypothesis testing problem as follows

(18) $$\begin{array}{c} {\tilde{H}}_{0}:u_{k}^{\left(i\right)}\in T\left(\eta \right)\;\;\;\;\left(\mathrm{noise}\right)\\ {\tilde{H}}_{1}:u_{k}^{\left(i\right)}\in T\left(s\right)\;\;\;\;\left(\mathrm{signal}\right) \end{array}$$

where ${\tilde{H}}_{0}$ and ${\tilde{H}}_{1}$, respectively, denote the transformed null and alternate hypothesis while T(s) denotes multiscale true signal coefficients or the multiscale version of *signal only* case and T(η) are multiscale noise (only) coefficients.

Based on proposed hypothesis testing problem for multiscale denoising in Equation (18), foundations of the *multiscale detection theory* can be built. To this end, a scale adaptive threshold $\lambda_{k}$ may be obtained by fixing the probability of a false alarm at the *k*th scale, i.e., $P_{fa}^{\left(k\right)}=\alpha^{\left(k\right)}$. Now a test statistic $S(u_{k}^{\left(i\right)})$ may be employed to compute the statistical distance between the multiscale coefficients $u_{k}^{\left(i\right)}$ from the distribution of noise at multiple scales, i.e., distribution of T(η). Henceforth, the transformed hypothesis testing problem in Equation (18) can be re-written as follows

(19) $$\begin{array}{c} {\tilde{H}}_{0}:S\left(u_{k}^{\left(i\right)}\right)\leq \lambda_{k};\;\;\;\;\;i.e.,\;\;\;\;\;u_{k}^{\left(i\right)}\in T\left(\eta \right)\\ {\tilde{H}}_{1}:S\left(u_{k}^{\left(i\right)}\right)>\lambda_{k};\;\;\;\;\;i.e.,\;\;\;\;\;u_{k}^{\left(i\right)}\in T\left(s\right). \end{array}$$

**Remark** **1.** *Null and alternate hypotheses in the proposed approach correspond to the noise only and signal only detections at multiple scales (i.e., ${\tilde{H}}_{0}:T\left(\eta \right)$ & ${\tilde{H}}_{1}:T\left(s\right)$ respectively), whereas the null and alternate hypothesis in traditional detection problem correspond to noise only and signal plus noise detections at original signal (image) scale (i.e., detection of $H_{0}:\eta$ & $H_{1}:S+\eta$ respectively).*

### 5.3. Estimation of Threshold $\lambda_{k}$

As a consequence of the modified hypothesis testing problem in Equation (19), the definition of $P_{fa}^{\left(k\right)}$ is also modified accordingly, which directly follows from Equation (11) as

(20) $$P_{fa}^{\left(k\right)}=Prob\left(S\left(u_{k}^{\left(i\right)}\right)>\lambda_{k}\;|\;{\tilde{H}}_{0}\right)=\;\;\int_{\left\{u_{k}^{\left(i\right)}\;|\;S\left(u_{k}^{\left(i\right)}\right)>\lambda_{k}\right\}}p\left(u_{k}^{\left(i\right)}|\;{\tilde{H}}_{0}\right)du_{k}^{\left(i\right)},$$

where $\left\{u_{k}^{\left(i\right)}\;|\;S\left(u_{k}^{\left(i\right)}\right)>\lambda_{k}\right\}$ is the set of multiscale noise coefficients which are falsely detected as signal, i.e., the set of coefficients yielding false alarms.

In the proposed framework, the threshold $\lambda_{k}$ for each scale *k* is estimated using Equation (20) for a given probability of false alarm $P_{fa}^{\left(k\right)}$ at scale *k*. For that purpose, probability distribution function of noise coefficients at multiple scales $p(u_{k}^{\left(i\right)}|\;{\tilde{H}}_{0})$ is required. The challenge is that the T(·) might change input noise distribution, e.g., UWT does not retain the Gaussianity at multiple scales. As a result, the probability density function $p(u_{k}^{\left(i\right)}|\;{\tilde{H}}_{0})$ at scale *k* is obtained by taking the derivative of the empirically estimated null (cumulative) distribution function $F_{0}^{\left(k\right)}\left(t\right)$ at *k*th scale as follows

(21) $$p\left(u_{k}^{\left(i\right)}|\;{\tilde{H}}_{0}\right)=\frac{d}{dt}F_{0}^{\left(k\right)}\left(t\right),$$

where $\frac{d}{dt}$ denotes the first order difference in the discrete case. The empirical estimation of the null EDF $F_{0}^{\left(k\right)}\left(t\right)$ for a non-linear transform is discussed in the next section.

Similarly, the definition of $P_{d}$ changes to the following

(22) $$P_{d}^{\left(k\right)}=Prob\left(S\left(u_{k}^{\left(i\right)}\right)>\lambda_{k}\;|\;{\tilde{H}}_{1}\right)=\;\;\int_{\left\{u_{k}^{\left(i\right)}\;|\;S\left(u_{k}^{\left(i\right)}\right)>\lambda_{k}\right\}}p\left(u_{k}^{\left(i\right)}|\;{\tilde{H}}_{1}\right)du_{k}^{\left(i\right)},$$

where $p(u_{k}^{\left(i\right)}|\;{\tilde{H}}_{0})$ denotes the probability distribution function of multiscale noise T(η) and $p(u_{k}^{\left(i\right)}|\;{\tilde{H}}_{1})$ denotes the probability distribution function of multiscale true signal T(s).

In order to estimate $P_{d}^{\left(k\right)}$ from Equation (12), distribution model $p(u_{k}^{\left(i\right)}|\;{\tilde{H}}_{1})$ must be known a priori. One limitation of the extension of detection theory to multiscale denoising lies in the non-availability of a prior distribution model for multiscale signal coefficients. One exception to this could be the multiscale coefficients obtained from the DWT, which have been shown to follow heavy tailed exponential distributions [26]. However, a prior assumption for $p(u_{k}^{\left(i\right)}|\;{\tilde{H}}_{1})$ may not be possible for other transforms, which means that $P_{d}$ could not be easily computed for a general multiscale transform T(·). Consequently, the tradeoff between $P_{fa}$ and $P_{d}$ is fixed experimentally.

**Remark** **2.** *For a given noise distribution, the threshold estimation is performed only once.*

### 5.4. Multiscale GoF Statistics Estimation

In order to perform local hypothesis testing based on EDF, GoF test statistic $S(u_{k}^{\left(i\right)})$ must be estimated for each window of local coefficients $u_{k}^{\left(i\right)}$ at multiple scales. The statistic $S(u_{k}^{\left(i\right)})$ computes the distance between the EDF $F^{i}\left(t\right)$ of the window centered around the coefficient $u_{k}^{\left(i\right)}$ from the null distribution function $F_{0}^{\left(k\right)}$ at scale *k*. The computationally convenient expressions of Anderson Darling (AD) and Cramer Von Mises (CVM) statistics used within the proposed framework are given as follows

(23) $$S_{AD}\left(u_{k}^{\left(i\right)}\right)=-L-\sum_{j=1}^{L}\frac{\left(2j-1\right)}{L}\left(\ln\left(F_{0}^{\left(k\right)}\left(u_{k}^{\left(i\right)}\right)\right)-\ln\left(F_{0}^{\left(k\right)}\left(u_{k}^{\left(L+1-j\right)}\right)\right)\right).$$

(24) $$S_{CVM}\left(u_{k}^{\left(i\right)}\right)=\frac{1}{12L}+\sum_{j=1}^{L}\left((F_{0}^{\left(k\right)}\left(u_{k}^{\left(j\right)}\right)-\frac{\left(2j-1\right)}{L}\right).$$

For generality we denote both AD and CVM statistic by $S(u_{k}^{\left(i\right)})$.

As discussed above, the non-linear transforms change the distribution of noise at multiple scale, hence, to compute $S(u_{k}^{\left(i\right)})$ using Equations (23) and (24), $F_{0}^{\left(k\right)}\left(t\right)$ must be known a priori for each scale *k* for a given non-linear transform T(·). To this end, we estimate $F_{0}^{\left(k\right)}\left(t\right)$ empirically by assuming a large sized AWGN η which is subsequently decomposed using multiscale transform as follows ${\underline{u}}_{k}^{\left(i\right)}=T\left(\eta \right)$. Next, multiscale noise coefficients ${\underline{u}}_{k}^{\left(i\right)}$ at each scale *k* are divided into *M* windows of local coefficients centered at the spatial location i. Subsequently, EDF $F_{\eta }^{i}\left(t\right)$ of all windows is computed followed by the ensemble average of these EDFs resulting in the reference or null distribution $F_{0}^{\left(k\right)}\left(t\right)=Avg\left\{F_{\eta }^{i}\left(t\right)\right\}\;\forall \;i$.

**Figure 6:**
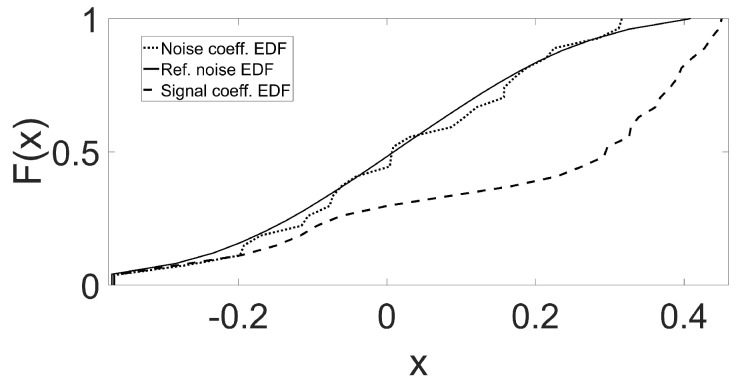
Hypothesis testing based signal and noise detection: $F_{0}^{\left(k\right)}\left(t\right)$ is reference Gaussian CDF plotted along with of $F_{s}^{i}\left(t\right)$ of signal coefficients (dashed line) and the EDF $F_{\eta }^{i}\left(t\right)$ of noise coefficients (dotted line).

To give an insight into the GoF based hypothesis testing, Figure 6 plots the null distribution $F_{0}^{\left(k\right)}\left(t\right)$ of a DTCWT scale (solid line) along with the EDF $F_{s}^{i}\left(t\right)$ of signal coefficients (dashed line) as well as the EDF $F_{\eta }^{i}\left(t\right)$ of noise coefficients (dotted line). As $F_{0}^{\left(k\right)}\left(t\right)$ and $F_{s}^{i}\left(t\right)$ are far apart, the statistic $S(u_{k}^{\left(i\right)})$ is expected to be quite large in this case and consequently suggests alternate hypothesis (detection of signal). Contrarily, $F_{0}^{\left(k\right)}\left(t\right)$ and $F_{\eta }^{i}\left(t\right)$ are very close suggesting that $S(u_{k}^{\left(i\right)})$ corresponds to noise.

### 5.5. Multiscale Thresholding Based on Hypothesis Testing

For a threshold $\lambda_{k}$ obtained for given $P_{fa}=\alpha$ in Equation (20), the following hard thresholding function is employed based on the proposed hypothesis testing in Equation (19) for all the windows of multiscale coefficients corresponding to noisy signal

(25) $${\hat{u}}_{k}^{\left(i\right)}=\left\{\begin{array}{cc} 0 & \;if\;\;S\left(u_{k}^{\left(i\right)}\right)\;\leq \;\lambda_{k},\\ u_{k}^{\left(i\right)} & \;if\;\;S\left(u_{k}^{\left(i\right)}\right)\;>\;\lambda_{k}, \end{array}\right.$$

where the central coefficient $u_{k}^{\left(i\right)}$ is replaced by zero if null hypothesis is fulfilled, i.e., $S\left(u_{k}^{\left(i\right)}\right)\;\leq \;\lambda_{k}$; otherwise $u_{k}^{\left(i\right)}$ is retained yielding the thresholded coefficients ${\hat{u}}_{k}^{\left(i\right)}$.

In order to obtain the denoised image, the thresholded multiscale coefficients from Equation (25) are reconstructed by employing the transform $T^{-1}\left(\cdot \right)$ as follows

(26) $$\hat{S}=T^{-1}\left({\hat{u}}_{k}^{\left(i\right)}\right),$$

where $\hat{s}$ an estimate of the true signal (or image) s, or simply stated the denoised signal or image. Implementation of various forward and inverse wavelet transforms is reported in [54,55,56,57].

### 5.6. Inverse VST

In the case of Poisson denoing, exact unbiased inverse of Anscombe transform (Inv-AT) [20] of the $\hat{S}$ is performed to obtain denoised image $\hat{\tilde{S}}$. Similarly for Poisson–Gaussian denoising, inverse generalized Anscombe transformation (Inv-GAT) performed on the Gaussian denoised $\hat{S}$ to obtain the Poisson–Gaussian denoised image ${\hat{\tilde{S}}}^{{}^{\prime }}$.

## 6. Experimental Results

**Figure 7:**
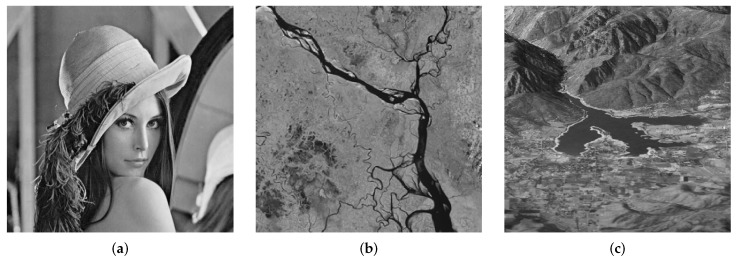
Input (2D) signals or images used for experimentation in this work including (**a**) ‘Lena’ image, (**b**) ‘Padma River’ image and (**c**) ‘Ogden Valley’ image.

This section presents image denoising results of proposed methods and gives comparison with respect to the state-of-the-art. To that end, we choose to employ undecimated wavelet transform (UWT) and dual tree complex wavelet transform (DTCWT) as transform domain methods within the proposed framework. The test statistics include AD and CVM for hypothesis testing within the framework of the GoF test. The input images used in this study include standard test images ‘Lena’, ‘Plane’, ‘Peppers’ and ‘Boat’ while two images, respectively, capturing aerial views of ‘Padma River’ and ‘Ogden Valley’ are obtained from on-line repository https://earthobservatory.nasa.gov/, which freely allows use, publication and redistribution of images. Figure 7 shows ‘Lena’ (https://en.wikipedia.org/wiki/File:Lenna_(test_image).png), ‘Padma River’ and ‘Ogden Valley’ while standard images such as ‘Boat’, ‘Plane’ and ‘Peppers’ are provided as supporting material with the manuscript, see the Figure S1 in the Supplementary Materials. We report quantitative denoising performance using the peak signal to noise ratio (PSNR), where each reported PSNR value is the average of J=20 iterations. Furthermore, a representative denoising example of a CMOS image corrupted with real noise is demonstrated where the real noisy image is obtained from the *RENOIR* dataset introduced in [58].

### 6.1. Poisson Denoising

In this section, we discuss the performance of the proposed Poisson denoising method against the state-of-the-art. For Poisson denoising using the proposed framework, UWT and DTCWT were employed as transform domain methods while AD statistics was used as test statistic within the GoF framework. We compare the proposed Poisson denoising methods against *MSVST* [28], *NLPCA* [18], *PureLet* [14] and *Poiss-NLM* [17]. The set of input test images is composed of standard ‘Lena’, ‘Plane’, ‘Peppers’ and ‘Boat’ images along with two images capturing the aerial view of ‘Padma River’ and ‘Ogden Valley’. These images were corrupted by signal-dependent Poisson noise arising from CMOS/CCD sensors where Poisson noise with varying intensities was added to the input images to simulate sensor noise. Since noise here is signal-dependent, increasing the peak amplitude of signal, increases the peak signal to noise ratio (PSNR) of the noisy image.

Each input image was corrupted by Poisson noise at varying signal peaks, i.e., 1–100 and the resulting input PSNR values are listed in Table 1, along with output PSNR values obtained by denoising these images using the state-of-the-art and the proposed Poisson denoising methods. Results in Table 1 show that the proposed *AT-AD-DTCWT* method yielded highest output PSNR values on most instances when compared to the other methods. The proposed *AT-AD-UWT* method also demonstrated comparable performance by consistently yielding second or third highest output PSNR values while at times it also managed to outperform all of the comparative methods. *PureLet*, which is considered as the gold standard method in Poisson denoising, remained competitive against the proposed methods and managed to beat them at a few input noise levels. *Poiss-NLM* also showed comparable denoising results but it mostly remained behind the *PureLet* and *AT-AD-DTCWT* methods in terms of the output PSNR values. *MSVST* failed to match the performance of the best methods but showed good denoising performance. *NLPCA* shows competitive performance on higher noise levels but as the noise level was reduced in the signal, *NLPCA* failed to improve its performance for all images.

**Figure 8:**
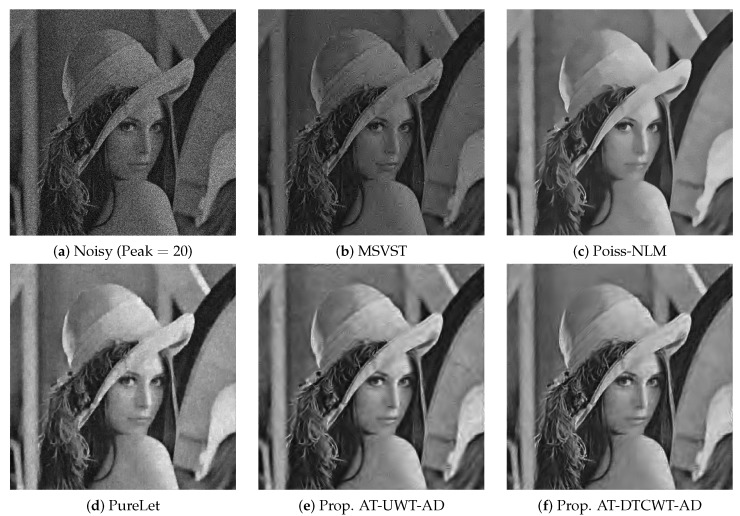
Poisson denoising results on the ‘Lena’ image by various methods at signal peak =20.

**Figure 9:**
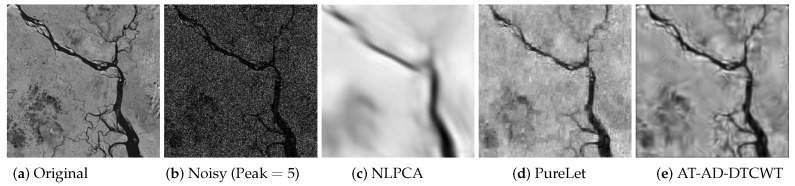
Poisson denoising results on the ‘Padma River’ image by various methods at signal peak =5.

We also display denoised images of ‘Lena’ and ‘Padma River’, respectively, in Figure 8 and Figure 9. In Figure 8, noisy ‘Lena’ image at signal peak =20 is displayed in Figure 8a along with the denoised ‘Lena’ images by *MSVST*, *Poiss. NLM*, *PureLet*, *AT-AD-UWT* and *AT-AD-DTCWT*, respectively, in Figure 8b–f. Observe from Figure 8d that *PureLet* yielded blurry artifacts which were spread all around the denoised ‘Lena’ image. Figure 8c shows the denoised signal by the *Poiss-NLM* which seems to be devoid of the artifacts but at the cost of the loss of image details, due to over-smoothing of the denoised signal. The denoised signals by the proposed methods *AT-AD-UWT* and *AT-AD-DTCWT*, shown in Figure 8e,f respectively, showed lesser artifacts as compared to the *PureLet* and the *MSVST* while also extracting more signal details. When compared to *Poiss-NLM*, denoised images by the proposed *AT-AD-DTCWT* method extracted more details but at the expense of slight artifacts. The *AT-AD-UWT* also extracted higher signal details compared to the *Poiss-NLM* but with some visible artifacts. *PureLet* and *MSVST* also changed the brightness of the denoised images whereas the proposed methods did not alter signal brightness even at such higher noise level.

Figure 9 compares the denoising performance of the proposed *AT-AD-DTCWT* method against the *PureLet* and the *NLPCA* at very high noise level, i.e., signal peak =5, on ’Padma River’ image. Original and noisy versions of ‘Padma River’ image are shown in Figure 9a,b while the denoised images from *NLPCA*, *PureLet* and the proposed *AT-AD-DTCWT* are shown in Figure 9c–e respectively. *NLPCA* not only over-smoothed the recovered image details but also blurred it, see Figure 9c. *PureLet* and the proposed *AT-AD-DTCWT* managed to recover significant image details even at such a high level of signal-dependent additive noise, see Figure 9d,e. Note that *Purelet* showed significant blurring artifacts in Figure 9d while denoised image by the proposed *AT-AD-DTCWT* recovered image details effectively with very little artifacts even at such a high input noise level, see Figure 9e.

### 6.2. Poisson–Gaussian denoising

We now provide comparative results of the proposed framework for Poisson–Gaussian denoising against the existing denoising methods. We use DTCWT as a transform domain method in the proposed methodology while *AD* is used as test statistic for the GoF based hypothesis testing. We name the proposed method *GAT-AD-DTCWT* and compare it against the *PGureLet* [15], *GAT-BLSGSM* [24] and *MSVST-MPG* [27]. We report the denoising results on all input images used in previous two sections, namely, ‘Lena’, ‘Plane’, ‘Peppers’, ‘Boat’, ‘Padma River’ and ‘Ogden Valley’. These images were corrupted with input Poisson–Gaussian noise of varying noise levels to simulate CMOS/CCD sensor noise where the strength of the Poisson noise was defined by signal peak = 1, 2, 3, 4, 5, and 10 and standard deviation σ of Gaussian noise was selected as σ=peak/10.

Input PSNR values corresponding to these parameters of Poisson–Gaussian noise are reported in Table 2, along with the output PSNR values of the denoised images by the comparative methods. Note that denoised images from the proposed method had highest output PSNR values on most input noise levels while the *GAT-BLSGSM* yielded competitive results. It was observed that *PGureLet* showed competitive results on higher noise levels while the *GAT-BLSGSM* yielded competitive results on lower noise levels. However, the proposed *GAT-AD-DTCWT* showed consistently improved performance at all noise levels. *MSVST-MPG* yielded lowest output PSNR values among all the methods.

**Figure 10:**
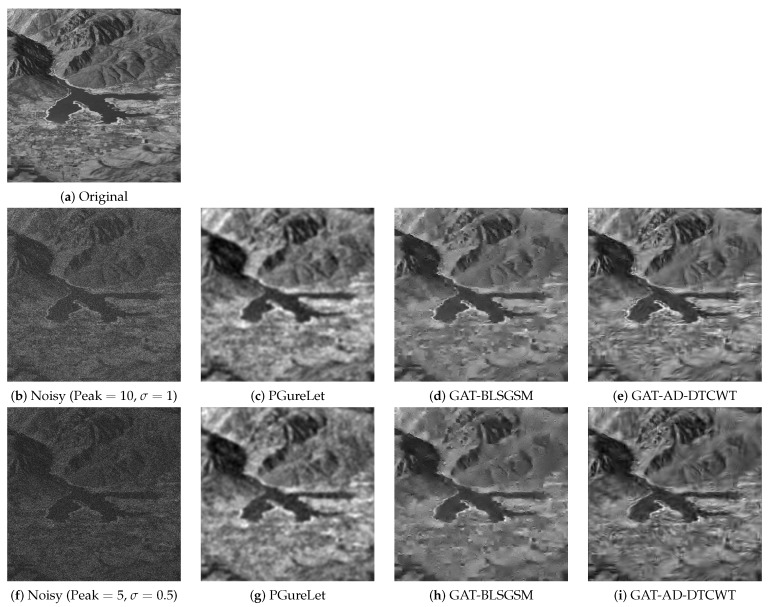
Poisson–Gaussian denoising results on the ‘Ogden Valley’ image by various methods where noisy image is corrupted by Poisson noise at signal peak =10 & σ=1 (second row) and at signal peak =5 & σ=0.5 (third row).

Figure 10 shows original, noisy and denoised ‘Ogden Valley’ images obtained from the comparative methods. Noisy images displayed in Figure 10b,f were, respectively, corrupted by Poisson–Gaussian noise at signal peak =5 and 10 and AWGN standard deviation σ=0.5&1. These noisy images were denoised using *PGureLet*, *GAT-BLSGSM* and proposed *GAT-AD-DTCWT* method which are, respectively, displayed in second, third and fourth columns of Figure 10. Observe that the *PGureLet* blurred the recovered images and distorted the information, see Figure 10c,g. *GAT-BLSGSM* recovered the image details well (see Figure 10d,h) as compared to the denoised images from *PGureLet*. However, denoised images by the *GAT-BLSGSM* yielded spike-like artifacts which are more evident in denoising at higher input noise level, as shown in Figure 10h. Denoised images by the proposed *GAT-AD-DTCWT* method are displayed in Figure 10e,i where not only image details have been preserved well but also the contrast and bright information is intact.

### 6.3. A Denoising Example of an Image Obtained from CMOS Sensor

In this section, we employ proposed *GAT-AD-DTCWT* to suppress real CMOS sensor noise from an image obtained through a CMOS camera installed in Xiomi Mi3 mobile. This image is made freely available as part of the RENOIR dataset [58] containing CMOS images corrupted by sensor noise. The study in [58] not only offers a noisy dataset but also compares the performance of the state-of-the-art Poisson and Poisson–Gaussian denoising methods for removing the sensor noise.

**Figure 11:**
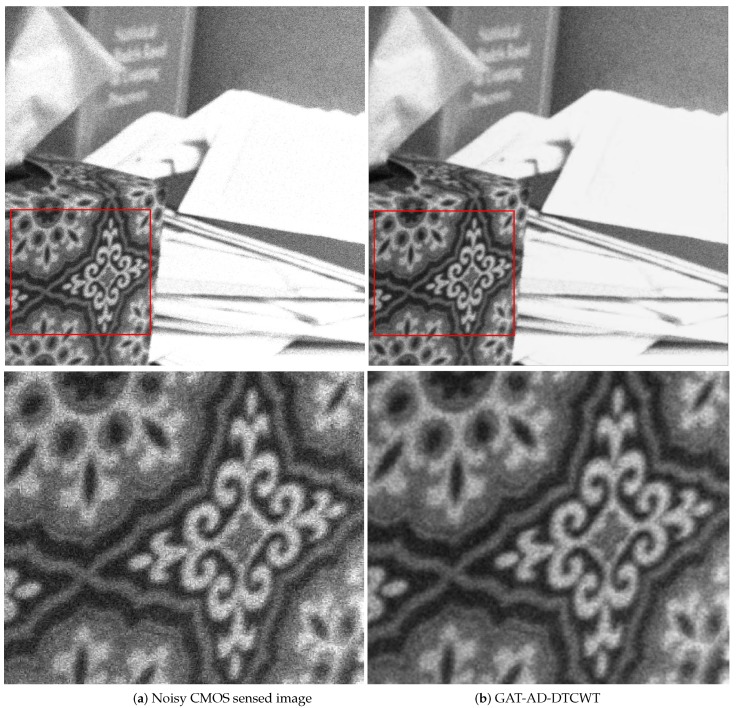
Performance analysis of the proposed *GAT-AD-DTCWT* on a noisy image obtained from RENOIR dataset [58] which contains noisy images from CMOS sensors corrupted by real sensor noise.

Figure 11 displays the noisy CMOS image along with the denoised image using the *GAT-AD-DTCWT* method. Noisy image (top row) and the zoomed in view of the highlighted part (lower row) are, respectively, shown in Figure 11a, while the denoised image using the *GAT-AD-DTCWT* (top row) and the zoomed in view of the highlighted region (lower row) in Figure 11b. As can be observed from the Figure 11 (top row) that the proposed method successfully suppresses majority of the real CMOS sensor noise. The zoomed-in view of the highly detailed region in the noisy and the denoised image are shown in Figure 11 (lower row), where granular noise pattern or shot noise spikes are visible in the noisy image patch. However, in the denoised image patch, these granular patterns have been successfully suppressed by the proposed method.

## 7. Discussion and Conclusions

This article proposes a generalized denoising framework based on detection theory and applies it to remove Poisson and Poisson–Gaussian noise from CMOS/CCD image sensors. To this end, variance stability transformation (VST) has been combined with the proposed binary hypothesis testing framework to enable the detection and removal of Poisson and Poisson–Gaussian noise at multiple scales. For local hypothesis testing, statistical measures of goodness-of-fit test based on empirical distribution function (EDF), i.e., Anderson Darling (AD), Cramer Von Mises (CVM) have been employed in our work. Furthermore, different 2D transform domain methods have been tested within the proposed framework.

The proposed methodology has been shown to outperform the comparative state-of-the-art methods in Poisson and Poisson–Gaussian denoising. This could be attributed to the effectiveness of the proposed framework to handle the non-standard noise distributions due to its data driven nature. To further stress this point, an example of denoising a CMOS image corrupted with real sensor noise using the proposed *GAT-AD-DTCWT* is also presented which demonstrates the efficacy of the proposed framework for suppressing the noise due to CMOS/CCD sensors.

Computational complexity of the proposed framework can be minimized by offline estimation of thresholds versus $P_{fa}$ table which is only required to be computed only once. Similarly, the estimation of the reference CDF (in case of nonlinear transformation) may be also be performed offline. Other computationally intensive step involved in the proposed method is the local estimation of the EDF which requires computations of the order of O(LlogL) while rest of the steps in the proposed methodology require computations equivalent to standard multiscale denoising methods.

The scope of this work is limited to the algorithm design for denoising CMOS/CCD images. However, in the following, we discuss few important aspects related to the hardware implementation of the proposed method. The proposed algorithm has a potential to be implemented in six stage-pipelined architecture, which enables parallel computations and increases the throughput of the system in real-time. The first-pipelined stage applies VST as a pre-processing step on the input noisy signal. The computationally expensive part of this stage is to evaluate square-root, CORDIC algorithms are used to compute square-root in hardware platforms (e.g., microcontrollers, processors and FPGAs). The second-pipelined stage computes the transform (UWT/DTCWT) operation on the pre-processed signal of the first stage. Real-time implementations of different transforms are reported in the literature [54,55,56,57]. Addition and average mathematical operations are needed to be executed in the third-pipelined stage of the proposed algorithm. In addition, these operations are executed in windowing fashion, therefore different blocks can be executed in parallel. In the fourth-pipelined stage, hard thresholding is done using GOF thresholding technique, which can be done by comparator implementation. The fifth-pipelined stage computed inverse transform operation, which has same computational complexity as the forward transform. Sixth-pipelined stage computed the exact inverse VST which requires pre-computed tables to empirically remove the bias at low photon count as stated in [20,25]. These tables can be stored and used in look-up table to decrease the computational complexity of this stage.

## Figures and Tables

**Table 1:** Poisson denoising: Output PSNR value obtained from the proposed method and state-of-the-art comparative methods for input images at varying input PSNR levels (bold denotes highest PSNR).

Peaks	Inp.	MS-	NL-	Pure-	Pois-	AT-AD	AT-AD	Inp.	MS	NL	Pure-	Pois-	AT-AD	AT-AD
	PSNR	VST	PCA	Let	NLM	UWT	DTCWT	PSNR	VST	PCA	Let	NLM	UWT	DTCWT
	**Lena**							**Plane**						
1	2.93	17.19	20.67	22.08	19.73	21.65	**22.22**	1.11	14.62	19.06	20.53	17.33	19.80	**20.45**
2	5.96	17.70	20.87	**23.31**	21.40	23.02	23.26	4.15	14.61	19.09	21.68	19.34	21.34	**21.70**
3	7.73	17.86	20.74	**23.98**	22.64	23.85	23.87	5.90	14.86	19.18	22.56	20.71	22.41	**22.77**
4	8.95	18.04	20.77	24.51	23.57	24.73	**24.86**	7.16	15.43	19.19	23.07	21.65	23.02	**23.32**
5	9.95	18.44	20.78	24.89	24.28	**25.67**	25.43	8.13	15.94	19.15	23.54	22.40	23.58	**23.96**
10	12.97	20.23	20.49	26.27	26.39	26.79	**27.18**	11.13	18.09	19.07	24.90	24.44	25.21	**25.58**
20	15.97	22.39	20.32	27.75	27.54	28.47	**28.84**	14.13	20.48	18.84	26.42	25.59	26.86	**27.29**
50	19.91	25.45	17.98	29.58	30.15	30.48	**30.75**	18.12	23.65	18.44	28.48	28.20	29.09	**29.49**
100	22.95	27.78	19.53	31.28	32.23	32.07	**32.32**	21.13	26.04	18.73	30.14	30.42	30.74	**31.23**
	**Peppers**							**Boat**						
1	2.73	16.61	18.82	**21.45**	19.28	20.96	21.44	2.96	16.83	20.17	21.28	19.06	20.72	**21.36**
2	5.76	17.00	18.94	22.80	21.12	22.58	**22.80**	5.96	17.43	20.22	22.22	20.48	21.89	**22.24**
3	7.54	17.25	18.81	**23.55**	22.40	23.26	23.39	7.72	17.47	20.29	**22.77**	21.56	22.63	22.74
4	8.80	17.58	18.87	24.17	23.36	**24.22**	24.18	8.94	17.59	20.31	23.27	22.39	23.11	**23.44**
5	9.72	17.96	18.74	24.49	24.04	24.85	**24.89**	9.93	17.92	20.20	23.69	22.98	23.62	**23.86**
10	12.75	19.83	18.36	25.75	26.01	26.34	**26.38**	12.94	19.72	20.21	24.90	24.77	24.88	**25.23**
20	15.79	22.05	18.44	27.15	27.00	27.73	**27.81**	15.94	21.79	20.09	26.19	25.80	26.28	**26.72**
50	19.72	24.81	16.67	29.01	29.07	**29.37**	29.51	19.90	24.71	19.99	28.06	28.09	28.14	**28.67**
100	22.76	26.87	16.63	30.33	**30.61**	30.35	30.55	22.96	26.85	19.81	29.78	30.07	29.66	**30.22**
	**Padma River**							**Ogden Valley**						
1	3.27	16.71	**19.63**	19.91	18.66	19.48	19.58	4.01	18.12	20.81	**21.78**	20.13	21.54	21.68
2	6.26	17.53	19.77	20.66	19.90	20.44	**20.70**	7.05	18.75	20.83	**22.66**	21.18	22.54	22.42
3	7.97	17.53	19.77	21.15	20.75	21.07	**21.17**	8.82	18.88	20.79	23.09	22.01	**23.16**	22.97
4	9.29	17.50	19.80	21.58	21.43	21.47	**21.61**	10.06	18.92	20.90	23.60	22.64	**23.61**	23.44
5	10.25	17.75	19.81	21.86	21.80	21.74	**21.90**	11.01	19.18	20.87	23.91	23.10	**23.96**	23.81
10	13.29	19.11	19.82	22.91	**23.11**	22.73	22.97	14.02	20.42	20.90	25.04	24.50	25.06	**25.15**
20	16.30	20.87	19.71	24.06	23.81	23.79	**24.10**	17.00	22.40	20.82	26.26	25.33	26.28	**26.58**
50	20.24	23.42	19.44	**25.92**	25.35	25.13	25.54	20.99	25.12	20.75	28.10	27.43	28.07	**28.63**
100	23.29	25.43	19.61	**27.56**	26.78	26.21	26.73	24.01	27.34	20.60	29.70	29.66	29.62	**30.28**

**Table 2:** Poisson–Gaussian denoising: Output PSNR value obtained from the proposed method and state-of-the-art comparative methods for input images at varying input PSNR levels (bold denotes highest PSNR).

Peaks	Noise	Inp.	MSVST	GAT-BL	PGure-	GAT-AD	Inp.	MSVST	GAT-BL	PGure-	GAT-AD	Inp.	MSVST	GAT-BL	PGure-	GAT-AD
	std. σ	PSNR	MPG	SGSM	Let	DTCWT	PSNR	MPG	SGSM	Let	DTCWT	PSNR	MPG	SGSM	Let	DTCWT
		**Lena**					**Plane**					**Lena**				
1	0.1	2.87	17.06	18.63	21.98	**22.09**	1.07	14.28	19.98	20.10	**20.63**	2.69	16.47	17.27	**21.45**	21.30
2	0.2	5.79	16.70	23.10	23.12	**23.30**	4.03	13.90	21.69	21.18	**21.71**	5.63	16.23	21.16	22.59	**22.82**
3	0.3	7.48	16.41	24.57	23.65	**24.00**	5.71	13.97	22.43	21.69	**22.64**	7.30	16.04	23.26	23.12	**23.53**
4	0.4	8.63	16.64	**24.96**	23.97	24.64	6.92	14.66	23.17	21.95	**23.23**	8.48	16.48	24.21	23.39	**24.25**
5	0.5	9.53	17.17	**25.16**	24.13	25.08	7.48	15.36	23.54	22.11	**23.68**	9.37	16.98	**24.91**	23.56	24.86
10	1	12.17	19.21	26.51	24.52	**26.58**	10.6	17.46	25.43	22.45	**25.51**	12.00	18.84	**26.53**	23.83	26.02
		**Boat**					**Padma River**					**Ogden Valley**				
1	0.1	2.84	16.64	18.32	21.10	**21.31**	3.21	16.80	18.86	**19.90**	19.71	3.90	17.96	19.16	**21.84**	21.62
2	0.2	5.78	16.45	21.22	21.93	**22.27**	6.09	16.36	20.57	20.44	**20.69**	6.79	17.82	22.41	22.54	**22.56**
3	0.3	7.47	16.14	22.58	22.27	**22.86**	7.79	16.00	**21.28**	20.72	20.97	8.48	17.29	**23.06**	23.01	22.86
4	0.4	8.63	16.38	23.24	22.46	**23.29**	8.93	16.20	21.27	20.84	**21.39**	9.62	17.26	23.21	23.13	**23.23**
5	0.5	9.52	16.91	23.61	22.60	**23.69**	9.83	16.64	21.85	20.91	**21.93**	10.48	17.61	**23.65**	23.35	23.54
10	1	12.15	18.69	**25.12**	22.86	24.88	12.43	18.14	**23.02**	21.04	22.59	13.04	19.31	24.52	23.62	**24.64**

## Supplementary Materials

The following are available online at http://www.mdpi.com/1424-8220/19/1/206/s1, Figure S1: Standard input images used for performance analysis of various denoising methods in this study including (a) ‘Peppers’ image, (b) ‘Boat’ image, (c) ‘Plane’ image.
