# Supplementary material for: A Multiscale Denoising Framework Using Detection Theory with Application to Images from CMOS/CCD Sensors
Source: Sensors (Basel). 2019 Jan 8;19(1):206. doi: 10.3390/s19010206 (PMC6338905; doi:10.3390/s19010206)
Supplement: Supplementary file 1 [file sensors-19-00206-s001.zip › sensors-397927-supplementary.pdf]

sensors

MDPI

Article

A Multiscale Denoising Framework using Detection Theory with Application to Images from CMOS/CCD Sensors

Khuram Naveed [1,*], Shoaib Ehsan [2], Klaus D. McDonald-Maier [2] and Naveed ur Rehman [1]

1 Department of Electrical and Computer Engineering, COMSATS University, Park Road, Islamabad 45550, Pakistan; naveed.rehman@comsats.edu.pk
2 School of Computer Science and Electronic Engineering, University of Essex, Colchester CO4 3SQ, UK; sehsan@essex.ac.uk (S.E.); kdm@essex.ac.uk (K.D.M.-M.)
* Correspondence: khurram.naveed@comsats.edu.pk; Tel.: +92-51-9049-223

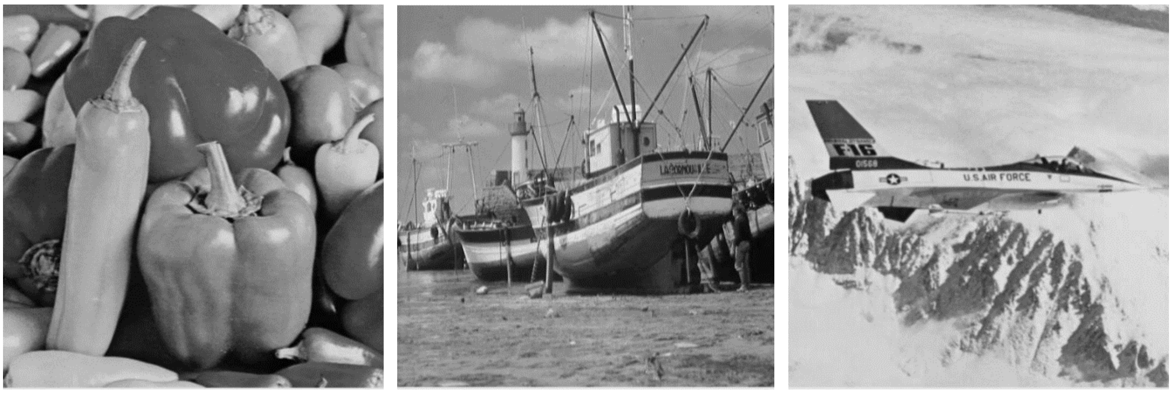

(**a**) (**b**) (**c**)

Figure S1. Standard input images used for performance analysis of various denoising methods in this study including (**a**) 'Peppers' image, (**b**) 'Boat' image, (**c**) 'Plane' image.
